# IL-1β Induces SOCS2 Expression in Human Dendritic Cells

**DOI:** 10.3390/ijms20235931

**Published:** 2019-11-25

**Authors:** Muamera Sarajlic, Theresa Neuper, Kim Tamara Föhrenbach Quiroz, Sara Michelini, Julia Vetter, Susanne Schaller, Jutta Horejs-Hoeck

**Affiliations:** 1 Department of Biosciences, University of Salzburg, 5020 Salzburg, Austria; muamera.sarajlic@sbg.ac.at (M.S.); theresa.neuper@sbg.ac.at (T.N.); foehrenbachki@stud.sbg.ac.at (K.T.F.Q.); sara.michelini@sbg.ac.at (S.M.); 2 Bioinformatics Research Group, University of Applied Sciences Upper Austria, 4232 Hagenberg im Muehlkreis, Austria; s1063406@stud.sbg.ac.at (J.V.); susanne.schaller@fh-hagenberg.at (S.S.); 3 Cancer Cluster Salzburg (CCS), 5020 Salzburg, Austria

**Keywords:** dendritic cell, IL-1β, SOCS2

## Abstract

Dendritic cells (DCs) regulate immunity and inflammation and respond to various stimuli, including cytokines. IL-1β is a key cytokine in the course of both acute and chronic inflammatory responses, making it indispensable for protection of the host, but also linking it to several diseases. Thus, IL-1β signaling must be tightly regulated. As suppressor of cytokine signaling (SOCS) proteins effectively control immune responses, we investigated the role of SOCS2 in IL-1β-induced DC activation. Human monocyte-derived DCs were stimulated with IL-1β, and SOCS2 mRNA and protein levels were measured. DC activation was assessed by cytokine secretion and surface marker expression. For functional analysis, small interfering RNA (siRNA)-based SOCS2 silencing was performed. SOCS2 expression was also analyzed in a curated NCBI GEO dataset of myeloid leukemia patients. We found IL-1β to be a potent inducer of SOCS2 expression. By silencing SOCS2, we showed that SOCS2 specifically limits IL-1β-induced IL-8 secretion. Moreover, our analysis revealed that SOCS2 levels are significantly increased in patients with acute and chronic myeloid leukemia, two hematological malignancies where disease progression is closely linked to IL-1β. This study identifies SOCS2 as a novel IL-1β-inducible target gene and points toward a potential role of SOCS2 in IL-1β-mediated DC activation.

## 1. Introduction

The interleukin-1 (IL-1) family of cytokines plays a key role in immunity, as it is critically involved in triggering inflammatory responses such as secretion of various cytokines and chemokines as well as increased production of nitric oxide and adhesion molecules. Thus, IL-1 family members are involved in innate and adaptive immune responses, and deregulation of IL-1 signaling is clearly linked to immunopathology. To date, eleven members of the IL-1 family have been described and can be divided into three subgroups (1): proteins with agonistic activity, including IL-1α, IL-1β, IL-18, IL-33, IL-36α, IL-36β, and IL-36γ; (2) the anti-inflammatory cytokine IL-37; and (3) the receptor antagonists IL-38, IL-1Ra, and IL-36Ra [1]. Since IL-1β is closely linked to a number of inflammatory diseases, it is the best studied member of the IL-1 family. In 1984, IL-1β was found to be primarily expressed as a precursor [2] requiring cleavage by caspase-1 to be biologically active [3,4]. Cleavage by caspase-1 and subsequent export of processed IL-1β were shown to be mediated by activation of the NLRP3 inflammasome [5]. To initiate an inflammatory response, IL-1β acts as ligand for IL-1 receptor 1 (IL-1R1) and its co-receptor IL-1R3, which are located at the cell membrane. Once IL-1β binds to IL-1R1, the two receptor chains combine to form a trimeric cytokine–receptor complex. This process brings the Toll–IL-1 receptor (TIR) domains into close proximity, which facilitates binding of the adaptor molecule MyD88 [6,7]. The subsequent interaction of MyD88 with IL-1R-associated kinases (IRAKs) [7,8] allows for binding of tumor necrosis factor-associated factor 6 (TRAF 6) [9], which in turn results in IL-1β-dependent activation of NF-κB [9], activator potein-1 (AP-1), extracellular signal-regulated kinases (ERK), p38 and other MAP kinases [10], c-Jun N-terminal kinase (JNK) and members of the interferon-regulatory factor (IRF) family [1]. This potent immune response turns IL-1β into an effective trigger of acute phase responses; however, uncontrolled IL-1β signaling can drive chronic non-resolved inflammation, which is often associated with a wide variety of human pathologies, including autoimmunity and cancer [11,12].

While IL-1α is constitutively expressed in a wide variety of cells, IL-1β is mainly expressed in myeloid cells, such as dendritic cells (DCs), and in turn also activates those cells [13]. Accordingly, IL-1β has been identified as a key cytokine in inflammation-related myeloid malignancies, including acute myeloid leukemia (AML) and chronic (CML) myeloid leukemia, as it supports cellular expansion and hence disease progression [14,15]. The relevance of DC-specific activation of the IL-1 signaling cascade was highlighted in a model of autoimmune myocarditis, in which engraftment of α-myosin-loaded IL-1R1^+/+^, but not IL-1R1^−/−^, DCs was capable of inducing inflammation in IL-1R1^−/−^ knockout mice [16]. However, information on the feedback regulation of IL-1β-induced signaling cascades in DCs remains scarce. It is well established that decoy receptors, the IL-1R antagonist (IL-1RA) and anti-inflammatory cytokines can control IL-1β-triggered inflammation [17], though little is known about other mechanisms that contribute to the regulation of IL-1β signaling in immune cells. IL-1 signaling and Toll-like receptor (TLR) signaling share various signaling molecules, which is why both are often referred to as members of the TIR (Toll/interleukin-1 receptor) family [18]. In human DCs, TLR signaling as well as NLR and cytokine signaling were shown to be modulated by the suppressor of cytokine signaling 2 (SOCS2) [19,20,21]. SOCS2 belongs to a family of intracellular proteins, which function as key regulators of innate and adaptive immunity by controlling cytokine-induced JAK/STAT activation as well as TLR-induced signaling events [22]. Thus, the present study aimed to investigate the potential role of SOCS2 in the IL-1β-induced signaling pathway in human DCs.

## 2. Results

### 2.1. IL-1β Induces SOCS2 Expression in Human Monocyte-Derived DCs

**Figure 1:**
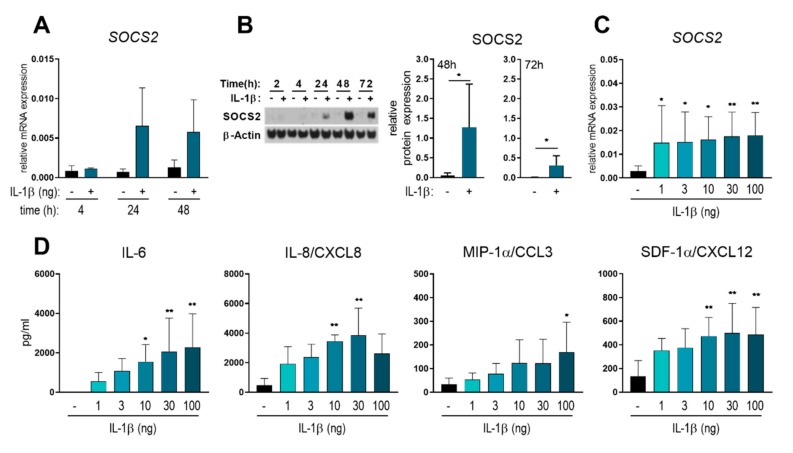
IL-1β triggers SOCS2 expression in human dendritic cells (DCs). Peripheral blood mononuclear cells (PBMCs) were isolated from buffy coats of healthy donors and monocytes were differentiated into monocyte-derived DCs (moDCs) for 6 days in the presence of IL-4 and GM-CSF. (**A**) Immature moDCs were stimulated with IL-1β (30 ng/mL) for 4, 24, or 48 h and SOCS2 mRNA was assessed by means of qPCR. Data represent mean + SD of four individual donors. (**B**) Time-dependent IL-1β-induced SOCS2 protein expression was detected by means of Western Blot and quantified after 48 and 72 h. Data represent mean + SD of at least five individual donors. For statistical analysis a two-tailed, paired Student‘s *t*-test was performed. (**C**,**D**) Immature moDCs were stimulated with increasing concentrations of IL-1β for 24 h and SOCS2 expression and cytokine secretion were detected by qPCR and multiplex assay, respectively. Data represent mean + SD of at least four independent donors. For statistical analysis, one-way ANOVA with Tukey’s post-hoc test was performed. * *p* < 0.05, ** *p* < 0.01.

To investigate whether SOCS2 might act as a negative feedback inhibitor of IL-1β signaling, we first monitored the ability of IL-1β to trigger SOCS2 expression. To do this, we analyzed SOCS2 mRNA as well as protein expression upon IL-1β stimulation in monocyte-derived DCs (moDCs) at several time points (Figure 1A,B). As previously observed for LPS-induced SOCS2 [19], IL-1β-induced SOCS2 mRNA expression is first detectable at 24 and 48 h post-stimulation (Figure 1A). Accordingly, we observed significantly enhanced SOCS2 protein levels at both those time points, peaking at 48 h post IL-1β stimulation; by 72 h, SOCS2 protein was clearly diminished (Figure 1B). We also investigated the dependence of SOCS2 expression on the concentration of IL-1β. Interestingly, we identified a significant increase in the level of SOCS2 mRNA even after stimulation with a low amount (1 ng/mL) of IL-1β, and this level of expression was not further enhanced by treatment with higher concentrations of IL-1β (Figure 1C). In contrast, the IL-1β-mediated secretion of pro-inflammatory cytokines and chemokines showed a concentration-dependent increase (Figure 1D). These data reveal that IL-1β is a potent inducer of both SOCS2 mRNA and protein expression in human moDCs.

### 2.2. Specific Effects of SOCS2 on IL-1β Signaling

**Figure 2:**
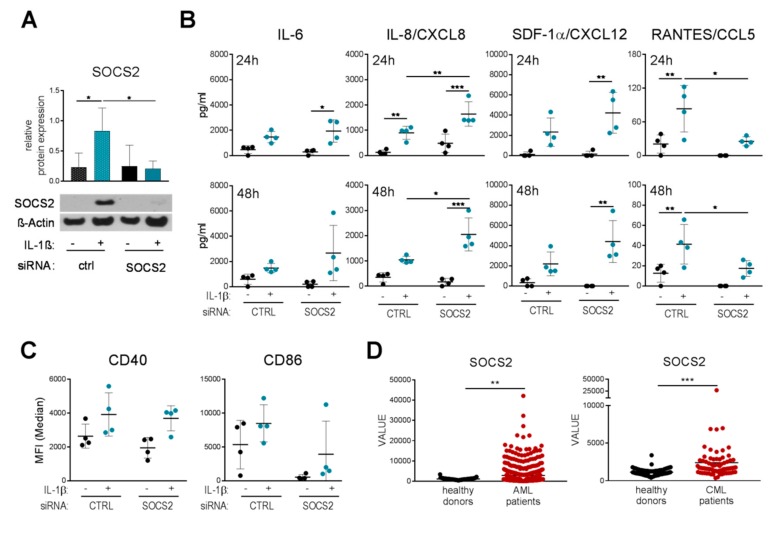
SOCS2 silencing enhances IL-1β-induced IL-8 and attenuates RANTES secretion in human DCs. On day 7 of differentiation, immature DCs were transfected with a non-targeting oligo or SOCS2-targeting small interfering RNA (siRNA; 100 pmol each) for 48 h; subsequently, DCs were stimulated with 30 ng/mL IL-1β for another 48 h. (**A**) Silencing efficiency was assessed by means of Western Blot analysis. Data represent mean + SD of five individual donors. For statistical analysis, one-way ANOVA with Tukey’s post-hoc test was performed. (**B**) Cytokine secretion of SOCS2-silenced DCs was analyzed 24 h or 48 h post IL-1β stimulation, respectively. (**C**) Surface marker expression was monitored by flow cytometry. Dots represent individual donors, lines indicate means ± SD. For statistical analysis, one-way ANOVA with Tukey’s post-hoc test was performed. (**D**) SOCS2 expression in acute myeloid leukemia (AML) or chronic myeloid leukemia (CML) patients as well as healthy donors was determined using a publicly available genomic dataset (GSE13159), which was analyzed using Python. For statistical analysis, a two-tailed, unpaired *t* test was performed. * *p* < 0.05, ** *p* < 0.01, *** *p* < 0.001.

SOCS proteins are known as negative feedback inhibitors; thus, members of the SOCS family suppress the same signaling pathways that previously activated their own transcription. Since we observed that IL-1β induces SOCS2, we next investigated whether SOCS2 inhibits IL-1β-induced DC maturation. Therefore, we performed RNA interference-based gene silencing with a small interfering RNA (siRNA) targeting SOCS2 or a non-targeting oligo and subsequently treated the cells with IL-1β. We then analyzed IL-1β-induced secretion of pro-inflammatory mediators as well as expression of co-stimulatory molecules. As shown in Figure 2A, SOCS2 protein expression was clearly decreased by SOCS2 silencing. Interestingly, analysis of cytokine and chemokine secretion revealed that IL-1β-induced production of IL-8 was significantly increased, whereas RANTES release was significantly decreased in absence of SOCS2. However, the secretion of all other tested mediators was not altered in moDCs lacking SOCS2 (Supplementary Materials Figure S1). Moreover, moDCs transfected with SOCS2 siRNA exhibited lower levels of CD86 compared to control cells, whereas CD40 levels were unchanged (Figure 2C). These data show that SOCS2 specifically inhibits IL-8 secretion, but not other cytokines, in response to IL-1β.

These specific effects of SOCS2 in the context of IL-1β are important because IL-1β-signaling is known to be associated with tumor progression in certain myeloid disorders such as acute myeloid leukemia (AML) and chronic myeloid leukemia (CML) [15]. Accordingly, we examined the expression levels of SOCS2 recorded in a publicly available gene expression dataset (NCBI GEO) for mononuclear cells collected from a panel of AML and CML patients. The results show significant upregulation of SOCS2 expression in AML and CML patients compared to healthy controls (Figure 2D), indicating that SOCS2 might play a role in those two myeloid malignancies.

## 3. Discussion

This study describes IL-1β as a potent trigger for SOCS2 expression in human moDCs. Analysis of IL-1β-induced SOCS2 expression over a time course of three days revealed that SOCS2 is stably expressed 24 h post IL-1β stimulation, peaks after 48 h and declines after 72 h. Interestingly, low amounts of IL-1β result in significantly enhanced SOCS2 expression after 24 h; however, SOCS2 levels are not further augmented upon stimulation with increasing concentrations of IL-1β. In contrast, IL-1β-dependent secretion of pro-inflammatory mediators increases in a concentration-dependent manner, suggesting that the molecular mechanisms promoting SOCS2 expression in IL-1β stimulated DCs might be distinct form those inducing the release of pro-inflammatory cytokines and chemokines. While NF-κB plays a key role in promoting the expression of pro-inflammatory genes, including numerous cytokines and chemokines in myeloid cells [23], this transcription factor seems to be dispensable for LPS-induced SOCS2 activation in human DCs [24]. Instead, the authors of the latter study suggest that SOCS2 is induced upon activation of an autocrine/paracrine loop involving the expression of type 1 interferons and subsequent activation of STAT3 and STAT5. That we observed SOCS2 protein expression no earlier than 24 h after IL-1β stimulation (Figure 1B) suggests that mediators induced via a secondary response might also be involved in IL-1β-induced SOCS2 expression.

As SOCS proteins are known as negative feedback inhibitors of cytokine signaling, we analyzed the effects of SOCS2 silencing on IL-1β-induced DC activation. While IL-8 secretion was significantly increased, RANTES release was attenuated in the absence of SOCS2; however all other tested cytokines/chemokines were only marginally affected by SOCS2 ablation. These data indicate that while SOCS2 is induced by IL-1β, it seems to primarily inhibit other signaling cascades, one of which could be the TLR4 pathway. We previously showed that, especially at later time points after TLR4 simulation, SOCS2 seems to control STAT3 activation, as silencing of SOCS2 leads to hyper-phosphorylation of STAT3, which in turn increases the expression of STAT3 target genes [19]; this indicates that IL-1β-induced SOCS2 may also be able to control TLR signaling. Moreover, a previous study by Hu et al. demonstrates reduced LPS-induced CD86 and CD40 expression upon SOCS2 silencing in human moDCs [25]. In the present study, we observed a similar trend, as SOCS2 silencing tended to decrease IL-1β-induced CD86, but not CD40, expression on DCs. As the signal transduction of the TLR pathway is highly comparable to that of the IL-1β pathway, these findings indicate that CD86 expression might be regulated via signaling molecules that are common to both TLR and IL-1β signaling, and can be controlled by SOCS2, whereas CD40 expression requires a different mechanism.

Additionally, we found that SOCS2 was differentially expressed in AML and CML patients compared to healthy subjects. It has been reported for CML that SOCS2 is overexpressed in advanced tumor stages and that SOCS2 expression is dependent on the Bcr-Abl mutation [26]. Moreover, SOCS2 has been described as one of four elements of a prognostic gene signature associated with AML, and a study on pediatric AML showed that high levels of SOCS2 correlate with poorer overall survival. In accordance, high SOCS2 expression was suggested to promote disease aggressiveness in AML patients [27,28]. Further investigations by Kazi et al. on the mechanism by which SOCS2 might support AML progression suggest that SOCS2 inhibits FLT3 signaling in AML by blocking Erk and STAT5 [29]. Interestingly, Radich and colleagues identified SOCS2 and IL-8 among the top ten genes associated with CML disease progression. However, they reported an inverse correlation of SOCS2 and IL-8, as they showed that SOCS2 is up-regulated whereas IL-8 is down-regulated in advanced CML compared to the chronic phase [30]. A recent study suggests that IL-8 as well as RANTES may also be valuable predictive markers in AML. The latter study reports that high serum IL-8 and low RANTES levels are correlated with a more favorable prognosis and may also be associated with a higher probability for AML patients to respond to immunotherapy [31]. In accordance, we detected significantly increased secretion of IL-8, but decreased RANTES expression in SOCS2-silenced, IL-1β-stimulated DCs. These studies clearly show that dysregulated SOCS2 expression can modulate the secretion of specific cytokines and is associated with leukemia; however, more studies are needed to fully understand the regulation and function of SOCS2 in leukemic cells. In addition, SOCS2 has also been identified to play a role in other cancer types such as melanoma, where specific loss of SOCS2 in DCs enhanced anti-tumoral immunity in a melanoma mouse model [32].

Taken together, the fact that SOCS2 is frequently deregulated in IL-1β-related cancers indicates that SOCS2 expression and its regulatory functions in DCs may support cancer progression by dampening anti-tumor immune responses.

## 4. Materials and Methods

### 4.1. Generation of Monocyte-Derived Dendritic Cells (iDCs)

This study was performed in accordance with the guidelines of the World Medical Association’s Declaration of Helsinki. No additional approval by the local ethics committee was required given that national regulations do not require informed consent in the case of anonymous blood cells discarded after plasmapheresis (buffy coats). Monocyte-derived DCs were differentiated from monocytes isolated from fresh buffy coats of healthy individuals (Blood Bank Salzburg, Salzburg, Austria) as described before [19]. In short, peripheral blood mononuclear cells (PBMCs) were isolated by density gradient centrifugation using Histopaque-1077 (Sigma-Aldrich, Vienna, Austria). After erythrocyte lysis with ACK buffer (150 mM NH_4_Cl, 10 mM KHCO_3_, 0.1 mM EDTA, pH 7.4), monocytes were separated by monocyte adherence (70 min at 37 °C, 5% CO_2_), and cultured in RPMI 1640 supplemented with 10% heat-inactivated fetal calf serum, 1% L-glutamine (Sigma-Aldrich), 10 U/mL penicillin/streptomycin (Sigma-Aldrich) and 50 µM β-mercaptoethanol (Gibco, Thermo Fisher Scientific, Darmstadt, Germany). Immature DCs were generated by addition of 50 ng/mL each of GM-CSF and IL-4 to the medium for 7 days. After two days of differentiation, fresh medium was added to the culture. After 7 days, DCs were harvested and seeded in 24-well plates for further experiments.

### 4.2. Flow Cytometry and Multiplex Assay

DCs were characterized by analysis of surface marker expression by using a FACS Canto II flow cytometer (BD Biosciences, San Francisco, USA) assessing median fluorescence intensities (MFI). Cells were harvested and resuspended in PBS before staining (30 min, 4 °C, dark) using the following antibodies: CD40-FITC (SC3) and CD86-PE (IT2.2) from eBioscience. Analysis was performed with FlowJo Software. IL-1β-induced cytokine and chemokine secretion (IL-6, IL-8/CXCL8, MIP-1α/CCL3, and SDF-1α/CXCL12) was assessed using the Cytokine/Chemokine/Growth Factor 45-Plex Human ProcartaPlex™ from ThermoFisher. In brief, beads were washed once (PBS, 0.05% Tween-20) and resuspended in assay buffer (PBS, 0.05% Tween-20, 1% heat-inactivated FCS). Of this mix, 8.34 µL were added to each well of a 96-well V-bottom format. Then, 15 µL of standard or samples were added and the plate was incubated on an orbital shaker at 4 °C overnight. The next day, samples were washed three times and resuspended in 15 µL of detection antibody solution and incubated for 30 min at room temperature. Samples were again washed three times before 20 µL of Streptavidin-PE solution (1:1 in assay buffer) were added to each well and incubated for 30 min at room temperature. Lastly, wells were washed three times and resuspended in drive fluid for analysis. Measurement was performed on a Luminex Magpix instrument and data were analyzed using Procarta Plex Analyst Software (Thermo Fisher Scientific, Darmstadt, Germany).

### 4.3. RNA Isolation and Quantitative Real-Time PCR

Total RNA was isolated using TRI Reagent (Sigma-Aldrich) and reverse-transcribed with RevertAid H Minus M-MulV reverse transcriptase (Thermo Fisher Scientific) according to the manufacturer’s instructions. Expression levels were determined by quantitative real-time PCR on a Rotorgene 3000 (Qiagen Instruments, Hombrechtikon, Switzerland) using IQ SYBR Green Supermix (BIO-RAD, Leipzig, Germany). Expression of the large ribosomal protein P0 (RPLP0) served as a reference gene. Relative mRNA expression x was calculated using the equation x = 2^−ΔCt^, where Δct represents the difference between the threshold cycle (ct) of the gene of interest and the reference gene. PCR specificity was assessed by recording a melting curve for the PCR products. The following primer pair was used for quantification: SOCS2: sense 5′-CCAAATCAACCAAAAAAAGTGACCATGAAGTCCTG-3′ and antisense 5′- CGGGGATTGAGTTGCACCTGTATAGCATGATATTC-3′.

### 4.4. Western Blot

Cell pellets of stimulated DCs were harvested and lysed in 60 µL of 2× Laemmli sample buffer (BIO-RAD, Leipzig, Germany) supplemented with 5% β-mercaptoethanol (Sigma-Aldrich). Subsequently, probes were separated on a 4%–12% gradient gel (NuPAGE, Life Technologies, Vienna, Austria) and blotted onto a nitrocellulose membrane (BIO-RAD), which was then blocked in TBS supplemented with 0.1% Tween and 5% nonfat dry milk for 1 h. The following antibodies were used according to the manufacturer’s instructions: SOCS2 (Cat no. 2779, Cell Signaling, Leiden, Netherlands) and HRP-linked anti-rabbit secondary antibody (Cat no. 7074S, Cell Signaling). Detection was done using West Pico PLUS Chemiluminescent Substrate (Thermo Fisher Scientific) and BioMax films (Kodak, Sigma-Aldrich, Vienna, Austria). Blots were quantified using ImageJ (NIH) software [33].

### 4.5. Gene Silencing via siRNA-Based Transfection

Transfection of DCs was performed using small interfering RNAs (siRNAs) targeting SOCS2 (InvitroGen, Thermo Fisher Scientific) or Allstars negative control (Qiagen, Hilden, Germany). For transfection, Lipofectamine RNAiMAX reagent (Life Technologies) was used according to the manufacturer’s instructions. In brief, 2 × 10^5^ DCs were seeded in 100 µL DC medium and transfected with 100 µL Opti-MEM (Life Technologies) containing 100 pmol siRNA and 1 µL transfection reagent. Thereafter, DCs were incubated for 6 h before 800 µL of fresh DC medium was added. After 48 h of incubation, DCs were stimulated for another 48 h and silencing efficiency was assessed by Western Blot analysis.

### 4.6. Database Analysis

The public genomic dataset GSE13159 from NCBI’s Gene Expression Omnibus (NCBI-GEO) [34] was used for analyzing IL-1β and SOCS2 expression in AML and CML patients and in apparently healthy individuals. The dataset used was part of the MILE Study (Microarray Innovations in Leukemia) program [35,36]. The study included whole-genome analysis data from 542 AML patients, 76 CML patients, and 74 healthy donors of a total sample size of 2096 from 11 participating centers on three continents. Analysis was performed using Python and the GEOparse package (https://github.com/guma44/GEOparse) for accessing and retrieving data from the GEO database. For the evaluations, the following Affymetrix Human Genome U133 Plus 2.0 Array Probe Set IDs in platform GPL570 were used: 203373_at for SOCS2.

### 4.7. Statistics

Data are presented as bars indicating mean ± standard deviation (SD) or as dot plots. Statistical analyses were performed with GraphPad Prism 7 software. Multiple groups were analyzed by one-way ANOVA including a post-hoc test. Two groups were analyzed by a paired Student’s *t*-test. *p* values <0.05 were considered significant (* *p* < 0.05, ** *p* < 0.01, *** *p* < 0.001).

## Supplementary Materials

Supplementary materials can be found at https://www.mdpi.com/1422-0067/20/23/5931/s1. Figure S1: The release of most tested cytokines and chemokines is not significantly affected by SOCS2 silencing.
