# Supplementary material for: IL-1β Induces SOCS2 Expression in Human Dendritic Cells
Source: Int J Mol Sci. 2019 Nov 25;20(23):5931. doi: 10.3390/ijms20235931 (PMC6928683; doi:10.3390/ijms20235931)
Supplement: Supplementary file 1 [file ijms-20-05931-s001.pdf]

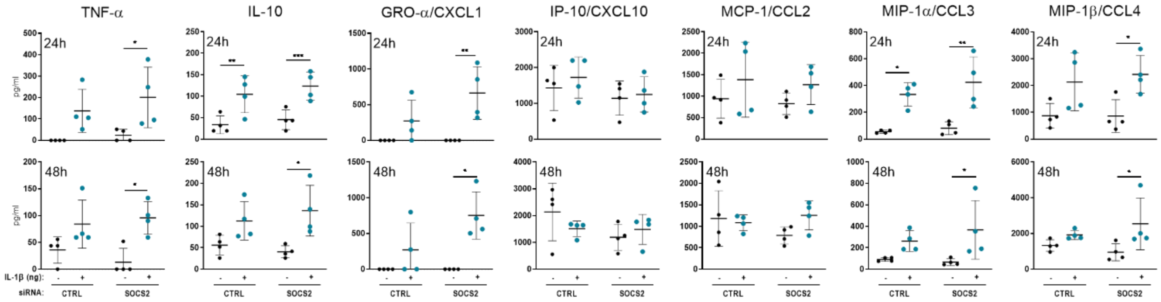


**Supplementary Figure 1. The release of most tested cytokines and chemokines is not significantly affected by SOCS2 silencing.** Immature DCs were transfected with a non-targeting oligo or SOCS2-targeting siRNA (100 pmol each) for 48 hours, followed by stimulation with 30 ng/ml IL-1β for another 48 hours. Cytokine secretion of SOCS2-silenced DCs was analyzed 24 hours or 48 hours post IL-1β stimulation, respectively (indicated). Dots represent individual donors, lines indicate means ± SD. For statistical analysis, one-way ANOVA with Tukey's post-hoc test was performed. *$p<0.05$, **$p<0.01$, ***$p<0.001$
